# Robotic assisted radical prostatectomy improves biochemical recurrence-free survival: The PROCA-life study

**DOI:** 10.1007/s11701-026-03157-3

**Published:** 2026-01-22

**Authors:** Tore Knutsen, Erling Aarsaether, Tom Wilsgaard, Martin Støyten, Einar Stikbakke, Elin Richardsen, Magnus Larsen, Inger Thune, Hege Sagstuen Haugnes

**Affiliations:** 1 Institute of Clinical Medicine, UIT- The Arctic University, 9037 Tromsø, Norway; 2 Department of Urology, University Hospital of North Norway, 9038 Tromsø, Norway; 3 Institute of Community Medicine, UIT- The Arctic University, 9037 Tromsø, Norway; 4 Department of Anesthesiology, University Hospital of North Norway, 9038 Tromsø, Norway; 5 Department of Oncology, University Hospital of North Norway, 9038 Tromsø, Norway; 6 Department of Pathology, University Hospital of North Norway, 9038 Tromsø, Norway; 7 Insitute of Clinical Medicine, University of Oslo, 0318 Oslo, Norway; 8 Department of Oncology, University Hospital of North Norway and The Arctic University, 9038 Tromsø, Norway

**Keywords:** Prostate cancer, Prostatectomy, Recurrence, Surgical margins

## Abstract

Robotic-assisted laparoscopic prostatectomy (RALP) is the preferred surgical technique compared to radical retropubic prostatectomy (RRP), but reports have been inconsistent on the effect of RALP on positive surgical margins (PSM) and biochemical recurrence (BCR). This study includes 420 men who participated in the population-based Tromsø study and PROCA-*life* study, diagnosed with prostate cancer (PCa) during 1995–2022, and treated with curative surgery. Overall, 171 PCa patients underwent open surgery with RRP, performed 1995–2012, and 249 PCa patients underwent RALP, performed 2012–2022. Preoperative and postoperative clinical characteristics were recorded. Multivariable Cox regression models presented with hazard ratios (HR) were used to study the association between surgical technique, preoperative and postoperative characteristics, and BCR. Overall, 72 patients (42.1%) in the RRP group, and 47 patients (18.9%) in the RALP group had BCR. Median time from surgery to BCR was 26.9 (IQR 10.2–58.0) months and 24.5 (IQR 15.4–46.9) months, respectively. PSM were three times more frequent in the RRP group compared to the RALP group (42.9% vs. 14.0%). Five-year BCR-free survival in the RRP group was 67% (95% CI 59–74), in the RALP group 81% (95% CI 75–86). The RALP procedure independently decreased the risk of BCR compared to RRP (HR 0.52; 95% CI 0.34–0.80, *p* = 0.003). PCa patients who had PSM had a doubled risk of BCR compared with patients with negative surgical margins (HR 2.00; 95% CI 1.31–3.05, *p* < 0.001). Patients with ISUP grade 3–5 (vs. grade 1) had an increased risk of BCR, highest with ISUP 4/5 (HR 3.44, 95% CI 1.79–6.61, *p* < 0.001). Implementation of RALP reduced the risk of biochemical recurrence compared to RRP, partly explained by the markedly lower PSM rate after RALP.

## Introduction

Treatment options for men with localized Prostate cancer (PCa) include radical prostatectomy or radiotherapy, and survival is presumably similar regardless of administered treatment [1]. Men with high-risk or locally advanced PCa have an increased risk of treatment failure and ultimately death from PCa, and there is a lack of consensus regarding the optimal treatment for these men [2]. In Norway, as many as 49% of men with incident localized PCa had radical prostatectomy as their primary curative treatment during 2004–2022 [3].

A biochemical recurrence (BCR), defined as rising prostatic-specific antigen (PSA), is expected to occur in 27%-53% of patients after radical prostatectomy [2]. Salvage radiotherapy (SRT) is the only curative option for men with BCR, and is recommended unless metastatic disease, contraindications to treatment or limited life expectancy [2]. Since the introduction of SRT in early 2000, the selection of patients expected to benefit from SRT has continuously been under debate, accompanied by improvements in diagnostic tools for excluding distant metastatic disease [2, 4]. Over the years, the documentation of long-term side effects of SRT include bowel, bladder and sexual complaints, and their impact on QOL, is increasing [5]. Due to the high incidence rate of BCR after prostatectomy, many European men are treated with SRT every year.

The association between PSM and an increased risk of BCR is well documented [6, 7], with a twofold increased risk of BCR with PSM and thus increased probability of SRT [8]. The effect of PSM on survival is debated, and other risk factors such as tumor grade and pathological T-stage may be equally important [9]. Of note, patient characteristics such as obesity may also influence the risk of BCR after prostatectomy [10]. Robotic-assisted laparoscopic prostatectomy (RALP) was gradually implemented during early 2000s [11], with advantages for post-operative complications, and in some studies, long-term lower rates of erectile dysfunction than with radical retropubic prostatectomy (RRP) [12–14]. Although RALP is the preferred surgical technique today in many countries, reports have been inconsistent on the effect of the RALP technique on PSM and the risk of BCR [12–15]. Consequently, there is a knowledge gap and a need for studies evaluating the impact of surgical technique on the risk of BCR [11].

The aim of this study was to evaluate whether the surgical technique (RALP vs. RRP) for men diagnosed with PCa treated during 1994–2022 had any influence on the risk of BCR. In addition, we have explored the associations between preoperative and postoperative clinical characteristics and risk of BCR.

## Materials and methods

### Study population

Among 22 647 males who participated in the population-based Tromsø study with high attendance rates [16], we included 17 542 men who participated in minimum one of the surveys Tromsø4 (1994–1995), Tromsø5 (2001), Tromsø6 (2007–2008) or Tromsø7 (2015–2016) in the PROCA-*life* study [17]. The procedures of invitations, screening and examinations were almost identical in these four surveys and the attendance proportions in the surveys varied between 66% and 75% [16, 18].

PCa cases in the Tromsø study were identified through linkage to the Cancer Registry of Norway by using the unique national 11-digit identification number. We used the National Population Registry of Norway and the Cause of Death Registry at the Norwegian Institute of Public Health to collect information on emigration and death.

**Fig. 1:**
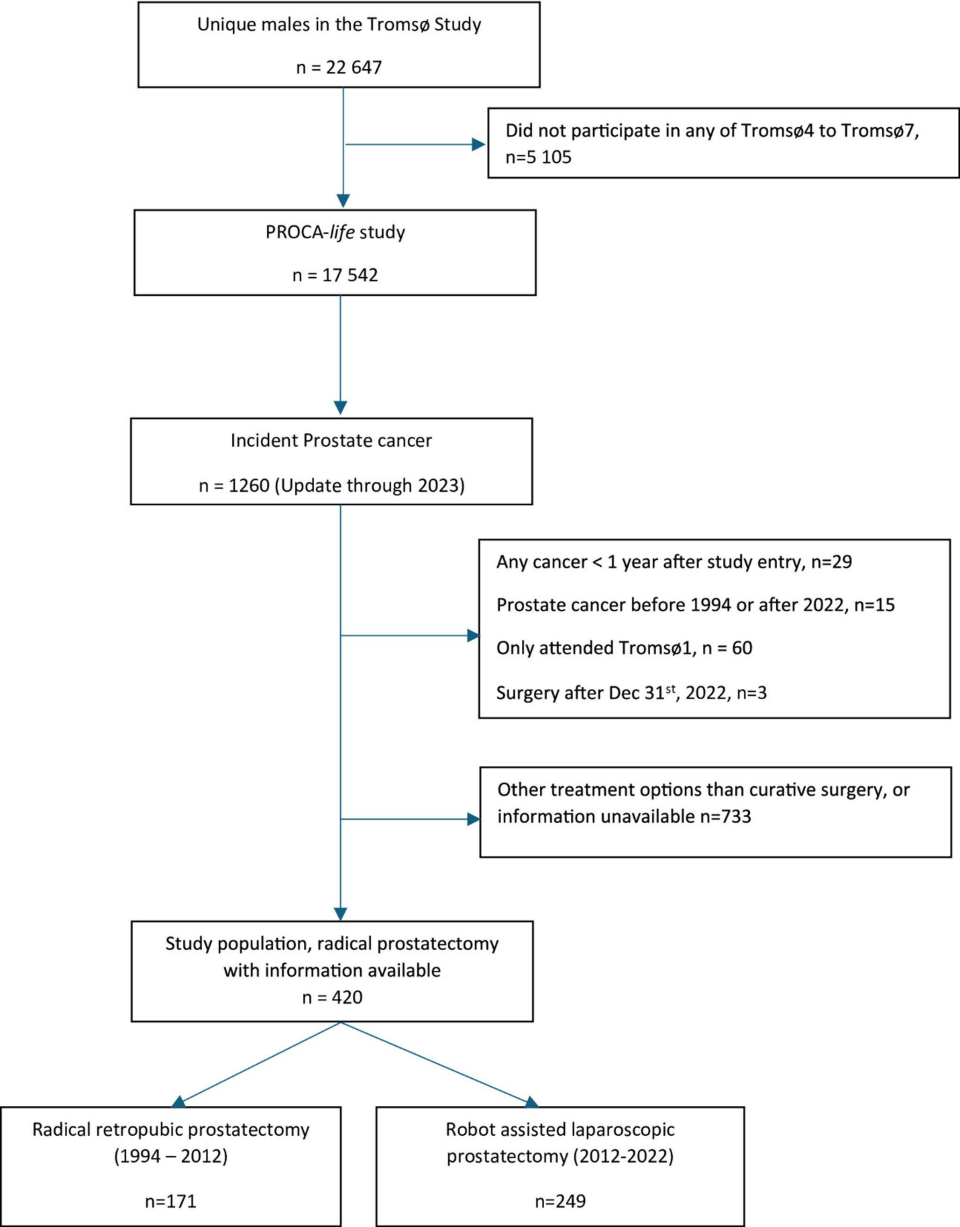
Flow chart for the study population. The PROCA-*life* study

A total of 1260 men developed PCa during the follow-up period from 1994 to 2022. Overall, 107 men were excluded due to any cancer diagnosis until one year after study entry (*n* = 29) or diagnosed with PCa before 1994 or after 2022 (*n* = 15), or other causes (*n* = 63) (Fig. 1). A total of 733 PCa patients had received either no curative treatment or non-surgical curative treatment, or clinical information was unavailable, and were excluded. Hence, 420 men diagnosed with prostate cancer and who had radical prostatectomy surgery with curative intent were included. A total of 171 PCa patients underwent open surgery with RRP, performed in the period 1994–2012, and 249 PCa patients underwent RALP, performed in the period 2012–2022.

### Questionnaires and clinical assessments

The participants in the Tromsø study responded to questionnaires which included medical history, lifestyle and socioeconomic factors [18].

A physical examination was performed by trained personnel. Body mass index (BMI) was calculated using the formula; weight (kg)/height^2^ (m^2^) and categorized in three groups with cut offs < 25.0 kg/m^2^ (normal weight), ≥ 25 kg/m^2^ - <30.0 kg/m^2^ (overweight), and ≥ 30 kg/m^2^ (obesity). Blood pressure (BP) was measured in a resting position, and the mean of the last two of three measurements was used. Smoking was categorized as current daily smoking or not. Physical activity was dichotomized as ≥ 3 exercise sessions pr week (physically active), or < 3 sessions pr week. Information on BMI, BP, smoking status and physical activity were included for participants at entry in the Tromsø study (preoperative clinical characteristics).

Blood samples were collected and analyzed by the Department of Laboratory Medicine, University Hospital of North Norway, Tromsø, Norway. PSA measurements were performed as part of clinical routine in diagnosis and follow-up (1990–1994 Stratus^®^ PSA Fluorometric Enzyme Immunoessay, 1994–2001 AxSYM Psa Reagent Pack, Abbot^®^, 2001-onwards Bayer^®^ PSA Reagens Pack Immuno I.

### Histopathological and clinical characteristics

Histopathological information of the PCa cases was obtained from histopathological records. All histopathological specimens were reexamined by the same experienced uro-pathologist (ER) and reclassified according to the latest International Society of Urological Pathology (ISUP) guidelines on Gleason Score and ISUP grade [19]. Information about prostate volume, PSM, extra-prostatic extension, seminal vesicle infiltration, perineural infiltration, ISUP grade and pT and pN stage were obtained from histopathologic reports.

Medical records with clinical data were obtained by trained physicians (TK, MS and ES). From the medical records, information on age, BMI and PSA at diagnosis were obtained, as well as information about surgical technique (RRP vs. RALP), date of surgery, lymph node dissection (yes/no) and nerve-sparing technique (yes/no). Information on any recurrence, BCR with recurrence date and whether treatment with SRT had been administered were also retrieved from medical records. No patients received neoadjuvant or adjuvant endocrine or cytotoxic treatment.

Follow-up time was calculated from the date of surgery to the date of BCR, emigration, death or end of follow-up (December 31st, 2023), whichever event came first.

### Surgical procedures

From 1994 to 2012, open surgery (RRP) was the standard procedure. When performed, lymph node dissection was limited to a standard pelvic template (sPLND) involving nodes along the external iliac vein and in the obturator fossa. Indication for sPLND was PSA at diagnosis ≥ 10 µg/l or ISUP grade 4 or 5 in biopsy specimen. In case of positive nodes on frozen section, the procedure was aborted.

In 2012, the RALP technique using the da Vinci^®^ system was introduced. During the period 2015–2017, we refined this technique by gradually implementing the retropubic suspension stitch [20]. Lymph node dissection during RALP followed an extended pelvic template (ePLND), including nodes caudal to the obturator nerve and medial/lateral to the internal iliac artery, bounded by the ureteral crossing cranially, the genitofemoral nerve laterally, the bladder medially and Cloquet’s node distally. Indication for ePNLD as part of the RALP procedure adhered to national and European guidelines (EAU) of the time, with ePLND as mandatory in high-risk patients, and recommended in intermediate-risk patients with ≥ 5% risk of lymph node metastasis according to validated nomograms.

Nerve-sparing technique (NS) was systematically implemented during the RALP era when deemed oncologically safe, and in line with published reports on technique and results [2]. During the RRP period, NS was not routine, although attempts to preserve neurovascular bundles were documented in 38% of cases, mainly in later years.

### Follow-up

The protocol for follow-up after RP was adjusted with the implementation of SRT as a curative option after BCR in 2003. Until 2003, PSA was measured after 3 and 6 months, and then annually. The main focus in the pre-SRT period was functional outcome and detecting clinical recurrence or metastasis.

With the implementation of SRT in 2003, an increasing focus on BCR was introduced during follow-up. PSA measurements have since been performed every 3 months for 2 years, and every 6 months for the following 3 years, thereafter annually. Due to increased patient volume and long travelling distances, PSA measurements after 3 months were performed by local health service, and patients referred to our institution if PSA was measured to ≥ 0.2 µg/l.

### Statistical analysis

The clinical characteristics of the study population are presented as means with standard deviation (SD), median with interquartile range (IQR), or percent with numbers. Differences between groups were compared using the χ^2^-test. Cumulative incidence curves with incidence of BCR were generated using the Kaplan-Meier method, with separate curves for surgical technique (RALP vs. RRP), surgical margin status, pT stage and ISUP grade in the surgical specimen.

Univariable and multivariable Cox regression models were used to estimate hazard ratios (HR) and 95% confidence intervals (95% CI) for the association between surgical technique, preoperative and postoperative clinical characteristics, and BCR (dependent variable). Postoperative characteristics tested in univariable analyses included prostate volume, nerve sparing technique, PSM, extra prostatic extension, seminal vesicle infiltration, pN and pT-stage of the surgical specimen, and ISUP grade. For a subset of the patients (*n* = 368), data on preoperative BMI categories, systolic and diastolic BP (continuous), smoking status and physical activity status were available and tested as independent variables in univariable Cox regression analyses.

To account for the impact of age, we used age as time scale in the BCR risk models. Based on potential biological mechanisms operating, the following variables from univariable analyses were included in the multivariable model: Surgical method (RALP vs. RRP), PSM, ISUP grade and pT-stage of the surgical specimen. The proportional hazard assumption was evaluated through graphical inspection of log-log curves and residual-based tests.

All statistical tests were two-sided with a significance level of 5%. Statistical analyses were conducted with STATA, version 17 (StataCorp. 2021. Stata: Release 17. Statistical Software. College Station, TX: StataCorp LLC).

### Ethical considerations

The PROCA-*life* has been approved by the Regional Committee for Medical and Health Research Ethics North (REK) (2015/1059), and the Tromsø study has been approved recently again by REK (2014/940 10.02.2015) and the Norwegian Data Protection Authority (Ref: 14/01463-4/CGN 02.03.2015). The PROCA-*life* research project is supported by the scientific committee of the Tromsø Study.

## Results

Among 420 PCa patients who had prostatectomy with curative intent, 171 patients underwent open surgery with RRP (41%), and 249 patients underwent RALP (59%). Median age at diagnosis was 63.1 years (IQR 59.2–67.9) and 65.0 years (IQR 60.3–69.8) for patients who received RRP and RALP, respectively (Table 1). The prevalence of obesity (BMI *≥* 30 kg/m^2^) at study entry was 11.7% in the RRP group, and 7.62% in the RALP group.

**Table 1:** Clinical characteristics of the participants at study entry and at time of radical prostatectomy according to surgical technique

Clinical Characteristic	RRP *N* = 171	RALP *N* = 249
At study entry:^1^		
Age at study entry, yrs	52 (47–56)	45 (40–50)
Categories of BMI, n (%)		
< 25 kg/m^2^	51 (35.2)	92 (41.3)
≥ 25 kg/m^2^ - <30 kg/m^2^	77 (53.1)	114 (51.1)
≥ 30 kg/m^2^	17 (11.7)	17 (7.62)
Blood pressure (BP), mmHg, mean (SD)		
Systolic BP Diastolic BP	138.8 (19.0) 83.1 (10.8)	134.7 (14.5) 79.6 (10.9)
Daily smoker, n (%)	41 (28.5)	53 (25.4)
Physically active, n (%)	58 (40.0)	105 (50.5)
At prostatectomy/diagnosis		
Age at diagnosis, yrs	63.1 (59.2–67.9)	65.0 (60.3–69.8)
BMI, kg/m^2^, mean (SD)^2^	27.2 (3.55)	26.9 (3.74)
PSA, µg/l, mean (SD)	11.9 (10.1)	10.3 (7.40)
ISUP Grade of biopsy, n (%)		
1	80 (51.6)	90 (36.6)
2	42 (27.1)	71 (28.9)
3	19 (12.3)	45 (18.3)
4 or 5	14 (9.0)	40(16.3)
Positive surgical margin, n (%)	73 (42.9)	34 (14.0)
Extraprostatic extension of tumor n (%)	52 (32.1)	76 (32.9)
Perineural infiltration	47 (27.8)	76 (32.6)
ISUP Grade of RP specimen n (%)^3^		
1	61 (37.0)	40 (16.3)
2	65 (39.4)	114 (46.5)
3	22 (13.3)	56 (22.9)
4 or 5	17 (10.3)	35 (14.3)
pT Stage of RP specimen n (%)		
pT2a	40 (24.2)	25 (10.3)
pT2b	16 (9.70)	13 (5.33)
pT2c	44 (26.7)	119 (48.8)
pT3a	39 (23.6)	57 (23.4)
pT3b or pT4	26 (15.8)	30 (12.3)

The PROCA-*life* study

Data are presented as median (IQR) unless otherwise specified. Numbers may vary due to missing values

Abbreviations: RRP, radical retropubic prostatectomy; RALP, robot-assisted laparoscopic prostatectomy; N, number; yrs, years; BMI, body mass index; SD, standard deviation; PSA, prostate-specific antigen; ISUP, International Society of Urological Pathology; RP, radical prostatectomy

^1^Overall, 52 patients entered the Tromsø study before survey T4 in 1994. General health characteristics are missing for these patients, leaving 368 men with available data

^2^BMI at diagnosis was available for overall 341 patients

^3^According to the 2014 guidelines from International Society of Urological Pathology [19]

Histopathological assessment of the surgical specimen observed that PSM were more frequent in the RRP group than in the RALP group (42.9% vs. 14.0%, *p* < 0,001). More patients in the RALP group than the RRP group had ISUP grade ≥ 3 (37.2% vs. 23.6%, *p* < 0.001), and pT-stage ≥ pT2c (84.5% vs. 66.1%, *p* < 0.001) (Table 1).

Median observation time in the RRP group was 6.23 years (IQR 1.96–14.4) in the RRP group, and 5.49 years (IQR 2.83–8.41 years) in the RALP group (Table 2). Overall, 42.1% of the patients experienced a BCR in the RRP group with a median time from surgery to BCR of 26.9 months (IQR 10.2–58.0). In total 18.9% of the patients in the RALP group had BCR with a median time from surgery to BCR 24.5 months (IQR 15.4–46.9). In total, 5 patients (1.19%) had persistently detectable PSA (PSA ≥ 0.2 µg/l) within 6 months after radical prostatectomy without A prior undetectable level (RALP 3 patients (1.21%), RRP 2 patients (1.17%).

**Table 2:** Observation times and characteristics of relapse after radical prostatectomy according to surgical technique, and for all included patients

Characteristic	RRP *N* = 171	RALP *N* = 249
Observation time, yrs^1^ Any recurrence, n (%) Biochemical recurrence, n (%) Treatment with salvage RT, n (%) Time from RP until recurrence, months^2^	6.23 (1.96–14.4) 84 (49.1) 72 (42.1) 35 (20.5) 26.9 (10.2–58.0)	5.49 (2.83–8.41) 62 (24.9) 47 (18.9) 15 (6.02) 24.5 (15.4–46.9)
5-year BCR-free survival, % (95% CI)	67 (59–74)	81 (75–86)

The PROCA-*life* study

Data are presented as median (IQR) unless otherwise specified. Numbers may vary due to some missing data

Abbreviations: RRP, radical retropubic prostatectomy; RALP, robot-assisted laparoscopic prostatectomy; N, number; yr, years; RT, radiotherapy; RP, radical prostatectomy; BCR, biochemical recurrence

^1^Observation time was calculated from time of RP until relapse, end of follow-up, emigration or death

^2^Calculated among patients with biochemical relapse

### Biochemical recurrence-free survival and risk factors for biochemical recurrence

**Fig. 2:**
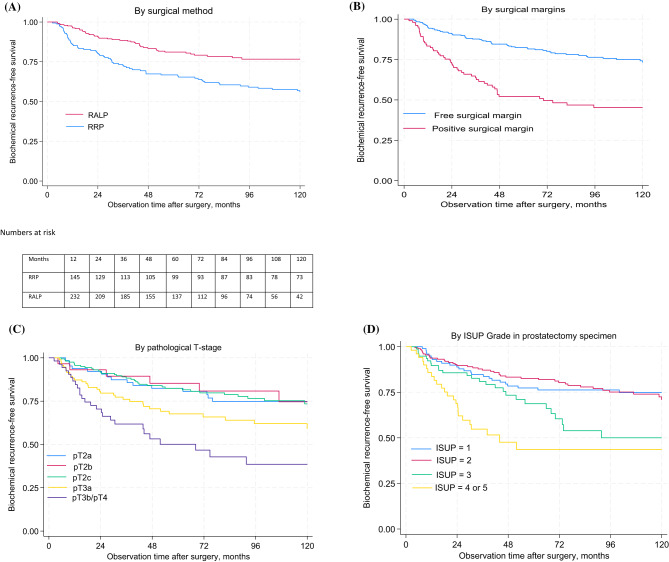
Kaplan-Meier plots for probability of biochemical recurrence-free survival after radical prostatectomy, by surgical method (**A**), presence of positive surgical margins in surgical specimen (**B**), pT-stage (**C**) and ISUP grade in prostatectomy specimen (**D**). Adjusted for age at surgery. The PROCA-*life* study. Abbreviations: RALP, robot-assisted laparoscopic prostatectomy; RRP, radical retropubic prostatectomy; ISUP, International Society of Urological Pathology

The 5-year BCR-free survival was overall 75% (95% CI 71% − 79%). Men who underwent RALP had a higher 5-year BCR-free survival in comparison to men operated with RRP (81% vs. 67%, p = < 0.001) (Table 2; Fig. 2A). The 5-year BCR-free survival was 83% among men operated with free surgical margins, compared with 53% among men with PSM (Table 2; Fig. 2B). A higher pT-stage or higher ISUP grade was associated with decreased probability of 5-year BCR-free survival and was lowest among men with pT3B or pT4 at 50%, and ISUP grade 4 or 5 in the surgical specimen at 44% (Table 2; Fig. 2C and D).

In univariable Cox regression models, preoperative health characteristics including BMI, systolic or diastolic BP, smoking or physical activity were not significantly associated with risk of BCR (Table 3). The RALP procedure was associated with a 54% reduced risk of BCR (HR 0.46, 95% CI 0.32–0.67, *p* < 0.001). Furthermore, PSM (HR 2.79, 95% CI 1.94–4.02, *p* < 0.001), ISUP grade ≥ 3 vs. ISUP grade 1, and pT stage ≥ pT3A vs. pT2A were associated with increased risk of BCR (Table 3).

**Table 3:** Univariable hazard ratios for the risk of biochemical recurrence after radical prostatectomy

Characteristics	*N*	HR (95% CI)	*p*-value
**Preoperative characteristics:**			
BMI at study entry, continuous BMI < 25 kg/m^2^ *(reference)* BMI ≥ 25 - <30 kg/m^2^ BMI ≥ 30 kg/m^2^ Systolic BP, continuous Diastolic BP, continuous Daily smoker Physically active	354 141 183 30 368 366 367 365	1.03 (0.97–1.10) 1.00 1.02 (0.64–1.63) 1.55 (0.89–2.69) 1.01 (1.00-1.02) 1.01 (0.99–1.03) 0.80 (0.52–1.23) 0.84 (0.58–1.23)	*0.369* *0.939* *0.120* *0.169* *0.215* *0.300* *0.374*
**Surgical characteristics:**			
Surgical method			
RRP *(reference)* RALP Prostate volume, continuous	171 249 347	1.00 0.46 (0.32–0.67) 1.00 (0.99–1.01)	*< 0.001* *0.440*
Nerve sparing technique			
No *(reference)* Yes	196 224	1.00 0.74 (0.52–1.07)	*0.107*
Lymph node dissection			
No *(reference)* Yes	216 195	1.00 1.48 (1.03–2.13)	*0.013*
Positive surgical margin			
No *(reference)* Yes	306 107	1.00 2.79 (1.94–4.02)	*< 0.001*
Extra prostatic extension			
No *(reference)* Yes	265 128	1.00 1.04 (0.93–1.16)	*0.531*
Seminal vesicle infiltration			
No *(reference)* Yes	348 51	1.00 1.05 (0.95–1.17)	*0.358*
Perineural infiltration			
No *(reference)* Yes	279 123	1.00 1.01 (0.92–1.10)	*0.823*
pN1 of RP specimen			
No *(reference)* Yes	166 28	1.00 1.63 (0.82–3.22)	*0.163*
ISUP Grade in RP specimen			
ISUP 1 *(reference)* ISUP 2 ISUP 3 ISUP 4 or 5	101 179 78 52	1.00 0.99 (0.61–1.62) 1.87 (1.08–3.23) 3.64 (2.06–6.43)	*0.974* *0.026* *< 0.001*
pT stage of RP specimen			
pT2A *(reference)* pT2B pT2C	65 29 163	1.00 0.92 (0.36–2.37) 1.08 (0.59–1.97)	*0.864* *0.807*
pT3A	96	1.87(1.01–3.47)	*0.045*
pT3B or pT4	56	3.61 (1.91–6.82)	*< 0.001*

The PROCA-*life* study

Abbreviations: N, numbers; HR; hazard ratio; CI, confidence interval; RRP, radical retropubic prostatectomy; RALP, robot-assisted laparoscopic prostatectomy; RP, radical prostatectomy; ISUP, International Society of Urological Pathology; BMI, body mass index; BP, blood pressure

In a multivariable Cox regression model, we observed that the RALP procedure had a 48% lower risk of BCR compared to RRP (HR 0.52, 95% CI 0.34–0.80, *p* = 0.003) (Table 4). Patients with PSM had a twofold higher risk of BCR (HR 2.00, 95% CI 1.32–3.05, *p* = 0.001), and among patients with ISUP grade ≥ 3 vs. ISUP grade 1, a higher risk of BCR was observed.

**Table 4:** Multivariable hazard ratios for the risk of biochemical recurrence after radical prostatectomy. The PROCA-*life* study

Characteristics	*N*	HR (95% CI)	*p*-value
Surgical method			
RRP *(reference)* RALP	171 249	1.00 0.52 (0.34–0.80)	0.003
Positive surgical margin			
No *(reference)* Yes	306 107	1.00 2.00 (1.31–3.05)	0.001
ISUP Grade in RP specimen			
ISUP 1 *(reference)* ISUP 2 ISUP 3 ISUP 4 or 5	101 179 78 52	1.00 1.09 (0.65–1.82) 2.32 (1.31–4.12) 3.44 (1.79–6.61)	0.747 0.004 < 0.001
pT stage of RP specimen			
pT2A *(reference)*	65	1.00	
pT2B pT2C pT3A pT3b or pT4	29 163 96 56	0.67 (0.26–1.75) 1.10 (0.59–2.08) 1.21 (0.63–2.36) 1.93 (0.95–3.92)	0.413 0.762 0.574 0.068

HRs are mutually adjusted for all listed variables using age time scale

Abbreviations: N, numbers; HR, hazard ratio; CI, confidence interval; RRP, radical retropubic prostatectomy; RALP, robot-assisted laparoscopic prostatectomy; RP, radical prostatectomy; ISUP, International Society of Urological Pathology

## Discussion

In this population-based study including 420 men diagnosed with PCa and operated with curatively intended prostatectomy during 1994–2022, the 5-year BCR-free survival was in favor of RALP in comparison to RRP (81% vs. 67%). Although men operated with RALP had more aggressive PCa in terms of higher ISUP grade and more advanced pT-stage than those who underwent RRP, they also had a lower frequency of positive margins (14% vs. 42.9%). Our data support a 48% reduced risk of BCR after RALP vs. RRP.

Our study compares BCR as an outcome after a surgical procedure that has evolved significantly during the study period. Even though the treatment principles are the same - surgical removal of the entire prostate gland with seminal vesicles - the differences in surgical technique are obvious. The RALP procedure gives the surgeon an improved visual control, resulting in less bleeding and better control of anatomical boundaries [12–14, 21]. This difference is so fundamental that the two groups in the study population cannot be compared unless this difference is addressed, either by exploring risk factors separately for the two subgroups, or by introducing the surgical method by itself as a covariate in the analyses, as we have done in the multivariable Cox regression model.

Importantly, PSM were independently associated with a twofold increased risk of BCR, consistent with previous studies [6–8]. Removal of tumors with ISUP grade ≥ 3 had a 2.3-3.4-fold increased risk of BCR compared with ISUP grade 1, in line with previous reports [7, 22]. In accordance with a recent metanalysis [23], we also observed an association between locally advanced disease (pT ≥ 3a) and a higher risk of BCR in univariable analysis. The markedly lower PSM rate after RALP compared with RRP (14.0% vs. 42.9%) likely represents an important explanation for the reduced BCR incidence in the RALP cohort. However, the RALP procedure had a significantly lower risk of BCR after adjustment for PSM, leaving the question of what protective effect this procedure inherits to be answered.

In the early study period, lymph node dissections were performed as sPLND. The patients operated in the later period, with RALP, had their lymph node dissections performed in accordance with updated recommendations, as ePLND [2]. Previous studies have shown a reduced risk of BCR after ePLND compared to sPLND, and also indications of improved metastasis-free survival after ePLND, but the literature is conflicting [24, 25]. During the early time period of the present study, sPLND was performed initially in the surgical procedure, awaiting frozen section of the lymph nodes, before continuing the surgery. If positive nodes were found on frozen section, the procedure was aborted, and the patient referred to medical treatment. This is reflected in the nodal yield from the two procedures, with 86% increase in number of excised nodes in ePLND compared to sPLND (mean 8.49, SD 4.47 vs. mean 15.8, SD 6.51, respectively). Lymph node dissections, and consequently, the nodal yield and pN-stage, are thus not suitable for comparisons between the two treatment groups. This is an important limitation herein, since microscopic lymph node metastases in situ despite a negative frozen section analysis in sPLND, could be an important contributor to the high proportion of BCR after RRP.

NS technique, as part of the RALP procedure, when applicable, could be expected to increase rates of PSM. In our study population, however, we noticed that the RALP procedure, despite a higher frequency of NS procedures, had a considerably reduced frequency of PSM.

The introduction of magnetic resonance imaging (MRI) as a standard preoperative workup [2], and subsequently the use of MRI-guided biopsies, might select patients for surgery with a lower risk of BCR. MRI of the prostate pre-biopsy was implemented as standard of care in Norway from 2015 [26], only a few years after the RALP technique was introduced in our institution. The risk of selection bias based on MRI-staging in our study is possible. However, the RALP group had more advanced pT-stage and more aggressive ISUP grade than the RRP group, contradicting the expectation that more advanced preoperative assessment in the later period would select candidates with lower risk of treatment failure.

The life expectancy is increasing among elderly men in many countries [27], with an increasing accept of curative interventions, including surgery, among older men. In our study, median age at diagnosis was 63.1 years for the RRP group, and 65.0 years for the RALP group, supporting this trend, and introducing a possible selection bias. The median time to BCR was 26.9 months for the RRP group, and 24.5 months for the RALP group. The median observation time was > 5 years in both treatment groups. Hence, a small difference in observation time does not explain the observed difference in BCR rate between RRP and RALP.

The most obvious advantage with the lower BCR rate after RALP vs. RRP is a reduced probability of consecutive SRT. Although SRT is an important curatively intended treatment option, it is associated with considerable long-term toxicity with a potentially reduced quality of life [5, 28–30]. In addition, SRT requires advanced technical equipment and represents a considerable load on health care systems, and thus, careful patient selection is important.

Our study has several strengths with a population-based approach, high attendance rate and completeness of identification of PCa due to mandatory registration of all new cases through the Cancer Registry of Norway (historically 98% complete) [31]. All medical records for the PCa patients were carefully reviewed by trained physicians with systematic abstraction of surgical and oncological treatment and BCR. We limited our study population in the present study to men without any previous history of cancer or PCa within the first year after study inclusion. Our cohort study is providing preoperative clinical characteristics such as obesity, which has been found to increase risk of PSM [32], hence with the possibility for increasing the risk of BCR.

There were also several limitations. The long study period inherently reflects changes in diagnostic workup, surgical expertise and perioperative management. Importantly, the number of cases per surgeon per year in our institution had a three-fold increase between 2000 and 2022 (data not shown), mirroring the national and international increase in prostate cancer incidence after the introduction of PSA-analysis. The implementation of RALP also parallelled the introduction of systematic training with simulators video recordings, allowing for detailed retrospective analysis of basically every surgical step of the RALP procedure, not available in the early period. Thus, it might be argued that the platform for surgical training and experience was more developed in the RALP vs. the RRP era, and this may in part explain some of our observed results. Finally, differences in lymph node dissection strategies limit direct comparison of nodal staging and may influence recurrence patterns. Although we adjusted for relevant clinical and pathological variables, residual confounding cannot be excluded.

In conclusion, we observed that implementation of RALP has reduced the risk of BCR with 48%, compared to previous RRP, despite larger and more aggressive tumors in the RALP group. The obvious benefit for the patients operated with the RALP technique is the reduced probability of consecutive SRT.

## Data Availability

The data are not available due to restrictions from the data protection officer and the Regional Committee for Medical and Research Ethics.
